# Development of 24-Hour Rhythms in Cortisol Secretion Across Infancy: A Systematic Review and Meta-Analysis of Individual Participant Data

**DOI:** 10.1210/clinem/dgae590

**Published:** 2024-08-29

**Authors:** Laura Kervezee, Michelle Romijn, Kirsten N G van de Weijer, Britney S J Chen, George L Burchell, Marieke S Tollenaar, Marcela Tamayo-Ortiz, Lauren E Philbrook, Carolina de Weerth, Yang Cao, Joost Rotteveel, Rina D Eiden, Rima Azar, Nicole R Bush, Adina Chis, Grazyna Kmita, Melissa W Clearfield, Roseriet Beijers, Michael Gröschl, Stefan A Wudy, Andries Kalsbeek, Evalotte Mörelius, Martijn J J Finken

**Affiliations:** Group of Circadian Medicine, Department of Cell and Chemical Biology, Leiden University Medical Center, 2333 ZC Leiden, The Netherlands; Department of Pediatric Endocrinology, Emma Children's Hospital, Amsterdam UMC, 1105 AZ Amsterdam, The Netherlands; Amsterdam Reproduction & Development Research Institute, 1105 AZ Amsterdam, The Netherlands; Department of Pediatric Endocrinology, Emma Children's Hospital, Amsterdam UMC, 1105 AZ Amsterdam, The Netherlands; Department of Pediatric Endocrinology, Emma Children's Hospital, Amsterdam UMC, 1105 AZ Amsterdam, The Netherlands; Medical Library, Vrije Universiteit Amsterdam, 1081 HV Amsterdam, The Netherlands; Department of Clinical Psychology, Institute of Psychology, Leiden University, 2333 AK Leiden, The Netherlands; Department of Environmental Health Sciences, Columbia Center for Children's Environmental Health, Columbia University Mailman School of Public Health, New York, NY 10032, USA; Department of Psychological and Brain Sciences, Colgate University, Hamilton, NY 13346, USA; Department of Human Development and Family Studies, The Pennsylvania State University, University Park, PA 16802, USA; Radboud University Medical Centre, Donders Institute for Brain, Cognition and Behaviour, 6525 EN Nijmegen, The Netherlands; Clinical Epidemiology and Biostatistics, Department of Medical Sciences, Faculty of Medicine and Health, Örebro University, Örebro 70185, Sweden; Unit of Integrative Epidemiology, Institute of Environmental Medicine, Karolinska Institute, Stockholm 17177, Sweden; Unit of Integrative Epidemiology, Institute of Environmental Medicine, Karolinska Institute, Stockholm 17177, Sweden; Department of Psychology and the Social Science Research Institute, The Pennsylvania State University, University Park, PA 16801, USA; Psychobiology of Stress & Health Lab, Psychology Department, Mount Allison University, New Brunswick, E4L 1C7, Canada; Departments of Psychiatry and Pediatrics, University of California, San Francisco (UCSF), San Francisco, CA 94158, USA; Department of Molecular Sciences, “Iuliu Hațieganu” University of Medicine and Pharmacy, 400349 Cluj-Napoca, Romania; Cognitive Neuroscience Laboratory, Babes-Bolyai University, 400294 Cluj-Napoca, Romania; Department of Clinical Psychology of Child and Family, Faculty of Psychology, University of Warsaw, 00-183 Warsaw, Poland; Department of Early Psychological Intervention, Institute of Mother and Child, 01-211 Warsaw, Poland; Department of Psychology, Whitman College, Walla Walla, WA 99362, USA; Department of Developmental Psychology, Behavioral Science Institute, Radboud University, 6500 HB Nijmegen, The Netherlands; Department of Cognitive Neuroscience, Donders Institute for Brain, Cognition and Behavior, Radboud University Medical Center, 6500 HB Nijmegen, The Netherlands; Celerion Switzerland AG, 8320 Fehraltorf, Switzerland; Paediatric Endocrinology & Diabetology, Center of Child and Adolescent Medicine, Justus Liebig University, D-35392 Giessen, Germany; Department of Endocrinology and Metabolism, Amsterdam UMC, University of Amsterdam, 1105 AZ Amsterdam, The Netherlands; Netherlands Institute for Neuroscience (NIN), an Institute of the Royal Netherlands Academy of Arts and Sciences (KNAW), 1105 BA Amsterdam, The Netherlands; Laboratory of Endocrinology, Department of Laboratory Medicine, Amsterdam UMC, University of Amsterdam, 1105 AZ Amsterdam, The Netherlands; Amsterdam Gastroenterology Endocrinology Metabolism, Endocrinology, Metabolism and Nutrition, 1105 AZ Amsterdam, The Netherlands; Amsterdam Neuroscience, Cellular and Molecular Mechanisms, 1105 AZ Amsterdam, The Netherlands; Department of Health, Medicine and Caring Sciences, Linköping University, 58185 Linköping, Sweden; School of Nursing and Midwifery, Edith Cowan University, Joondalup, WA 6027, Australia; Department of Pediatric Endocrinology, Emma Children's Hospital, Amsterdam UMC, 1105 AZ Amsterdam, The Netherlands; Amsterdam Reproduction & Development Research Institute, 1105 AZ Amsterdam, The Netherlands

**Keywords:** cortisol, hydrocortisone, adrenal cortex hormones, pediatrics, infant, endocrinology, circadian rhythm, biological clocks, adrenal insufficiency

## Abstract

**Context:**

In adults, cortisol levels show a pronounced 24-hour rhythm with a peak in the early morning. It is unknown at what age this early-morning peak in cortisol emerges during infancy, hampering the establishment of optimal dosing regimens for hydrocortisone replacement therapy in infants with an inborn form of adrenal insufficiency.

**Objective:**

We aimed to characterize daily variation in salivary cortisol concentration across the first year of life.

**Methods:**

We conducted a systematic review followed by an individual participant data meta-analysis of studies reporting on spontaneous (ie, not stress-induced) salivary cortisol concentrations in healthy infants aged 0-1 year. A one-stage approach using linear mixed-effects modeling was used to determine the interaction between age and time of day on cortisol concentrations.

**Results:**

Through the systematic review, 54 eligible publications were identified, reporting on 29 177 cortisol observations. Individual participant data were obtained from 15 study cohorts, combining 17 079 cortisol measurements from 1904 infants. The morning/evening cortisol ratio increased significantly from 1.7 (95% CI: 1.3-2.1) at birth to 3.7 (95% CI: 3.0-4.5) at 6 to 9 months (*P* < .0001). Cosinor analysis using all available data revealed the gradual emergence of a 24-hour rhythm during infancy.

**Conclusion:**

The early-morning peak in cortisol secretion gradually emerges from birth onwards to form a stable morning/evening ratio from age 6 to 9 months. This might have implications for hydrocortisone replacement therapy in infants with an inborn form of adrenal insufficiency.

In adults, the secretion of cortisol follows a pronounced 24-hour rhythm, with a peak in the early morning and a trough around midnight (1). In adults and older children with adrenal insufficiency, a condition in which the adrenal cortex fails to mount an appropriate cortisol response to stress, hydrocortisone replacement therapy is titrated based on this physiological 24-hour rhythm in cortisol secretion (2). If hydrocortisone replacement therapy does not match the physiological pattern of cortisol secretion, signs of both undertreatment (ie, too little hydrocortisone) and overtreatment (ie, too much hydrocortisone) may emerge. Undertreatment predisposes to adrenal crises, and in congenital adrenal hyperplasia, also to androgen excess, while overtreatment carries cardiometabolic risks like obesity, hypertension, and insulin resistance (3).

However, inborn forms of adrenal insufficiency usually manifest shortly after birth, the most prevalent being congenital adrenal hyperplasia due to 21alpha-hydroxylase deficiency. To avoid undertreatment or overtreatment with hydrocortisone in infants with an inborn form of adrenal insufficiency, knowledge about the normal developmental trajectory of hypothalamic-pituitary-adrenal (HPA) axis rhythmicity is essential. However, it is currently not clear at what age the adult-type 24-hour rhythm is established (4). Some studies suggest that a 24-hour rhythm in HPA axis activity might already be present in fetuses, with data showing that markers of the fetal adrenal zone were higher in the afternoon than at other times of the day (5, 6). Recently, it was found that at age 1 month, some but not all, infants exhibited a biphasic cortisol peak (ie, with both an early-morning peak and an afternoon peak) (7), suggestive of the presence of a perinatal transition phase in HPA axis rhythmicity. In general, the best estimate based on current data is that an adult-type rhythm of cortisol secretion is established somewhere between 2 weeks and 9 months of age (8).

To more precisely characterize the developmental trajectory of the 24-hour rhythm in cortisol secretion, we conducted an individual participant data (IPD) meta-analysis of published studies that determined salivary cortisol concentrations in healthy infants (from birth to 1 year of age). Thereby, we create normative data and provide insight into potential dosing regimens for hydrocortisone replacement therapy across infancy.

## Methods

### Protocol Registration

This systematic review and meta-analysis was reported in accordance with the Preferred Reporting Items for Systematic Reviews and Meta-Analysis of individual participant data (PRISMA-IPD) guidelines (9) and was registered in the PROSPERO international prospective register of systematic reviews (CRD42022323631). The medical research ethics committee of VU University Medical Center (VUmc) approved the study (protocol number: METC VUmc 2021.0620).

### Search Strategy

A systematic search was performed in the databases PubMed, Embase.com, Clarivate Analytics/Web of Science Core Collection, and Cumulative Index to Nursing and Allied Health Literature (CINAHL). The timeframe within the databases was from inception to February 7, 2022, and searching was conducted by G.L.B. and M.J.J.F. The search included keywords and free text terms for (synonyms of) “*adrenal cortex hormones*” combined with (synonyms of) “*saliva*” combined with (synonyms of) “*children*”. A full overview of the search terms per database can be found in Supplemental Table S1A-S1D (10). No limitations on date or language were applied in the search.

### Eligibility Criteria

Evaluation of eligibility and data extraction were performed independently by 2 reviewers (B.C. and K.v.d.W.). Disagreements were solved by consensus or after adjudication by a third reviewer (M.J.J.F.).

Studies (longitudinal studies, cross-sectional studies, or randomized controlled trials) reporting on salivary cortisol concentrations in healthy infants aged 0-1 year were eligible for inclusion. Studies focusing exclusively on preterm infants, maltreated infants, or infants whose mothers used corticosteroids or hard drugs during pregnancy or postpartum, as well as studies published before 2000, due to likely expiration of the retention period for data sets, were not eligible for inclusion.

### Data Extraction

Data that were extracted from papers of eligible studies included: country, sample size, infant sex, age(s) at assessment, sampling protocol, collection method, type of cortisol assay, and measures of assay quality control (eg, inter-assay and intra-assay coefficients of variation, sensitivity).

### Scoring of Risk of Bias

All eligible studies were assessed for risk of bias by 2 independent reviewers using an adapted version of the Newcastle-Ottawa Scale fitted for the purpose of this review, displayed in Supplemental Table S2 (10). Again, conflicts were solved by consensus or after adjudication by a third reviewer (M.J.J.F.). A score of 4 or higher (out of 6) was considered as low risk of bias, a score of 2 or 3 as moderate risk of bias, and a score of 0 or 1 as high risk of bias. Risk of bias across studies was evaluated by separately evaluating the characteristics of studies providing IPD and those not providing IPD.

### Initial Contact With Investigators of Eligible Studies

Authors of eligible studies were approached to provide subject-level data regarding the cortisol concentration(s) found (in nmol/L), the age(s) at assessment, the time of sampling, and infant sex. For this purpose, an email was sent out to the corresponding author, and, in case of no response, after 2 to 3 months another email was sent out (with a maximum of 3). Corresponding authors were offered co-authorship on the current manuscript provided they were willing to contribute to drafting or critically reviewing the manuscript and approved the final version. All those who contributed data are authors on the current manuscript (1 author per study that contributed data).

For studies addressing cortisol reactivity following a particular stressor, we requested only the baseline data. For studies in which particular groups like preterm infants or maltreated infants were overrepresented, we requested only the data of the nonclinical participants.

### Study Outcomes

The main outcome was defined as the difference between morning (samples collected between 06:00 and 12:00) and evening (samples collected between 18:00 and 00:00) cortisol concentrations across different age categories (0 months, 1 month, 2 months, 3-5 months, 6-9 months, and 10-13 months). The main outcome was specified after receipt of the IPD but before statistical analysis commenced. This deviation from preregistration was based on the time-of-day distribution of the obtained IPD: a relatively low number of cortisol observations were collected during the nighttime (see “Results”). Therefore, it was decided to restrict the main analysis to a morning and evening window. The preregistered main outcome was included as an exploratory outcome, defined as the variation of cortisol concentration by time of day as a continuous circular variable (clock time on a 24-hour scale) across different age categories.

### Statistical Analysis

Data distributions of individual studies were visualized to evaluate IPD integrity prior to statistical analyses. Subsequently, an individual-level meta-analysis using a one-stage approach (11) was performed to address the study outcomes. All analyses were performed in R version 4.2.2. Results of statistical tests were considered statistically significant if the two-sided *P* value was below .05.

For the main analysis, linear mixed-effects models were fit to the data using the R packages lme4 (version 1.1-32) (12). The initial full model was specified with log-transformed cortisol concentrations as dependent variable, the interaction between time of day and age as fixed effects, sex as a covariate, participant as a random effect on the intercept, and study as a random effect on the intercept and time-of-day slope to account for clustering by study. For the main analysis, time of day was categorized as morning (samples collected between 06:00 and 12:00) and evening (samples collected between 18:00 and 00:00). Samples collected outside those time windows were excluded for this analysis. Age was categorized as 0 months, 1 month, 2 months, 3-5 months, 6-9 months, and 10-13 months. It was investigated if the full model could be simplified by comparing the fit of the full model to simplified models (models with a simpler random effect structure and without sex as a covariate) using likelihood ratio tests.

Upon specification of the final (simplified) model, the statistical significance of the interaction between age and time of day was determined using the R package lmerTest (version 3.1-3) (13). The R package emmeans (version 1.8.3) was used to obtain estimated marginal means of the (untransformed) salivary cortisol values by time-of-day categories (ie, morning vs evening) across the different age categories. In addition, post hoc pairwise comparisons were performed with the emmeans package to: (i) determine the difference between morning and evening cortisol concentrations at each age category; and (ii) assess whether the model-predicted ratio between morning and evening cortisol concentrations differ across age categories (using interaction contrasts). For post hoc tests, *P* values were adjusted for multiple testing with Sidak correction.

Sensitivity analyses were performed to evaluate the influence of individual studies on the overall results using a leave-one-study-out approach. The final model (as determined in the main analysis) was iteratively fitted to datasets from which one study was omitted. For each hold-out cohort, the statistical significance of the interaction between age category and time of day (morning vs evening) was determined and the model-predicted ratio between morning and evening cortisol concentrations at the different age categories was obtained.

To better visualize the development of the 24-hour rhythm in cortisol concentrations across the different age categories, exploratory analysis was performed using two-harmonic cosinor analysis (14) in a linear mixed-effects modeling framework with the log-transformed cortisol concentrations as dependent variable, the interaction between the cosinor terms and age categories as fixed effects, and participant and study as random effects on the intercept. For this analysis, all available data points were included. The statistical significance of the interaction between the cosinor terms and age was determined using likelihood ratio tests, and the model-predicted cosinor curves were visually displayed for each age category.

## Results

### Search Results

Supplemental Figure S1 (10) presents the flowchart of the inclusion process. The literature search yielded a total of 3320 citations. Of these, 1405 were unique records. Screening of titles and abstracts led to rejection of 1223 citations and review of 182 full-text articles, 54 of which met the eligibility criteria. In total, these 54 eligible publications reported on 29 177 cortisol observations.

Corresponding authors of 32 eligible articles either never responded (13 publications) or declined to participate (19 publications). Reasons for nonparticipation were not having access to data (11 publications), not being interested in participating (3 publications), local restrictions regarding data sharing (2 publications), and no longer having raw data (3 publications). Corresponding authors of 22 eligible articles were willing to send their data. From 6 of them we never received data, leaving the data of 16 publications, reporting on 15 unique cohorts, for IPD meta-analysis.

### Characteristics of Studies Included in IPD Meta-analysis

Table 1 presents the characteristics of the 15 unique cohorts included in the IPD meta-analysis (7, 15-29). These studies were conducted in North America and Europe, and their sample size ranged from 17 to 296 participants. In all, 17 079 observations collected from 1904 participants were available for IPD meta-analysis. The number of available observations was 867 for 0 months, 2542 for 1 month, 951 for 2 months, 3765 for 3-5 months, 3577 for 6-9 months, and 5377 for 10-13 months. The time of day at which samples were collected differed between studies, with some studies collecting samples throughout the entire 24-hour period, while others sampled at one or multiple fixed time points (see Supplemental Figure S2 (10) for an overview per study). Studies used a variety of collection methods, mostly swabs. All studies except for one (7) used immunoassay for cortisol analysis. Intra-assay and inter-assay coefficients of variation ranged from 3% to 15% in the 10 studies that reported these metrics. In 5 studies, no quality control data was reported (see Supplemental Table S3 (10)). Table 2 presents the risk of bias assessment of included studies, ranging from moderate to low. Visualizing the distribution of cortisol levels by study revealed a systematic error in the reported concentrations from 2 studies, presumably due to a unit conversion error from µg/dL to nmol/L. The cortisol values from these studies were corrected prior to statistical analysis.

**Table 1.** Characteristics of studies included in IPD meta-analysis

First author (publication year)	Study abbreviation*^a^*	Country (latitude)	N (% boys)	Total number of observations provided	Age(s) at sampling	Sampling protocol*^b^*	Collection method	Risk of bias assessment
Azar, et al (2010) (15)	AZ	Canada (44 °C)	188 (45%)	188	2-6 mo	Mid-morning sample	NR	Moderate
Beijers, et al (2016) (16) *^c^*	BE	The Netherlands (52 °C)	17 (34%)	85	5-7 wks	4 random samples on 2 different days	Dental eye sponges	Low
Cao, et al (2009) (17)	CA	USA, North Carolina (36 °C)	155 (50%)	359	0-12 mo	Random samples	NR	Moderate
Chis, et al (2017) (18)	CH	Romania (47 °C)	118 (46%)	118	< 2 hours	Sample obtained at <2 hours following birth	SalivaBio Infant's Swabs (Salimetrics, USA)	Moderate
Clearfield, et al (2014) (19)	CL	USA, Washington (46 °C)	32 (55%)	94	3 mo, 6-11 mo	3-point day curve	Salimetrics Infant Swab (Salimetrics, USA)	Low
Eiden, et al (2011) (20)	EI	USA, New York (43 °C)	189 (51%)	189	7-11 mo	Random samples	Dental cotton roll	Moderate
Gröschl, et al (2003) (21) *^d^*	GR	Germany (50 °C)	28 (NR)	84	0-5 mo, 8 mo, 10-11 mo	3-point day curve	Modified medical pacifiers (Büttner-Frank, GE)	Low
Hollanders, et al (2020) (7)	HO	The Netherlands (52 °C)	56 (60%)	311	1 mo	Samples obtained before every fed	SalivaBio Infant's Swabs (Salimetrics, USA)	Low
Ivars, et al (2015) (22)	IV	Sweden (58 °C)	134 (49%)	8593	2 days, 1 mo, 2 mo, 3 mo, 4 mo, 5 mo, 6 mo, 7 mo, 8 mo, 9 mo, 10 mo, 11 mo, 12 mo	3-point day curve on 2 consecutive days	NR	Low
Jones-Mason, et al (2018) (23)	JM	USA, California (38 °C)	132 (48%)	132	17 days to 4 mo	Random sample	Salimetrics Infant's Swabs (Salimetrics, USA)	Moderate
Kmita, et al (2011) (24)	KM	Poland (52 °C)	30 (52%)	95	0-1 mo, 3 mo, 5-6 mo, 11-14 mo	Mid-morning sample	Dental cotton roll	Moderate
Philbrook, et al (2014 & 2016) (25,26) *^c^*	PH	USA, Massachusetts (42 °C)	160 (48%)	1543	0-4 mo, 5-9 mo	3-point day curve	Filter paper	Low
Rosa-Parra et al (2018) (27)	RP	Mexico (19 °C)	216 (52%)	1664	11-12 mo	4-point day curve on 2 days	Salivettes with cotton braids (Sarstedt, GE)	Low
Simons et al (2015) (28)	SI	The Netherlands (52 °C)	153 (55%) *^d^*	989	11-13 mo	4-point day curve on 2 days within 1 week	Eye sponges (BD Visispeare, USA)	Low
Tollenaar et al (2010) (29)	TO	The Netherlands (52 °C)	296 (53%)	2635	1-2 mo, 3-6 mo, 9-13 mo	Mid-morning sample on 2 weekdays and 2 weekend days	Eye sponges or cotton rolls	Moderate

Abbreviations: ELISA, enzyme-linked immunosorbent assay; LC/MS-MS, liquid chromatography–tandem mass spectrometry; NR, not reported; RIA, radioimmunoassay.

^*a*^Abbreviated study name used in Supplemental Figures S2 and S3 (10).

^*b*^On single day, unless otherwise indicated.

^*c*^Observations excluded due to missing data on sampling time.

^*d*^Missing data on infant sex.

**Table 2.** Risk of bias assessment of studies included in IPD meta-analysis

Study	Selection		Outcome		Total score	Risk of bias assessment
	Representativeness of sample (max *)	Sample size (max *)	Assessment of outcome (max **)	Laboratory analysis (max **)		
Azar, et al (15)	—	*	—	*	** (2)	Moderate
Beijers, et al (16)	*	—	**	*	**** (4)	Low
Cao, et al (17)	*	*	—	*	*** (3)	Moderate
Chis, et al (18)	*	*	—	*	*** (3)	Moderate
Clearfield, et al (19)	*	—	**	*	**** (4)	Low
Eiden, et al (20)	*	*	—	*	*** (3)	Moderate
Gröschl, et al (21)	*	—	**	*	**** (4)	Low
Hollanders, et al (7)	*	*	**	**	****** (6)	Low
Ivars, et al (22)	*	*	**	*	***** (5)	Low
Jones-Mason, et al (23)	*	*	—	*	*** (3)	Moderate
Kmita, et al (24)	*	—	—	*	** (2)	Moderate
Philbrook, et al (25)	*	*	**	*	***** (5)	Low
Philbrook, et al (26)	*	*	**	*	***** (5)	Low
Rosa-Parra, et al (27)	*	*	**	*	***** (5)	Low
Simons, et al (28)	*	*	**	*	***** (5)	Low
Tollenaar, et al (29)	*	*	—	*	*** (3)	Moderate

### Characteristics of Eligible Studies Not Included in IPD Meta-Analysis

Supplemental Table S4 (10) presents the characteristics of the 38 eligible studies that were not included in the IPD meta-analysis (30-67). These studies, encompassing a total number of 12 098 observations, were conducted in North America, Europe, Australia, New Zealand, and South America. Eleven studies took saliva samples at least 3 times a day (30, 31, 38, 40, 46, 49, 62-64, 66, 67), one of which did so at multiple ages (38). Studies used a variety of collection methods, and 35 of them used immunoassay for cortisol analysis. The risk of bias ranged from moderate to low, as displayed in Supplemental Table S5 (10).

### IPD Meta-Analysis

**Figure 1.**
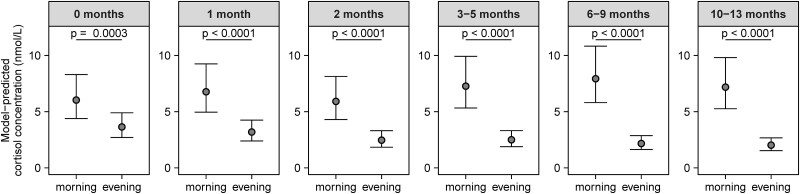
Salivary cortisol concentrations in the morning (06:00-12:00) and evening (18:00-00:00) across different age categories in early infancy. Data points are derived from the final main model (back-transformed from the log-scale) and shown as estimated marginal means and 95% CI. *P* values represent results from post hoc pairwise comparisons between morning and evening at the different age categories (adjusted for multiple correction using Sidak's method).

**Figure 2.**
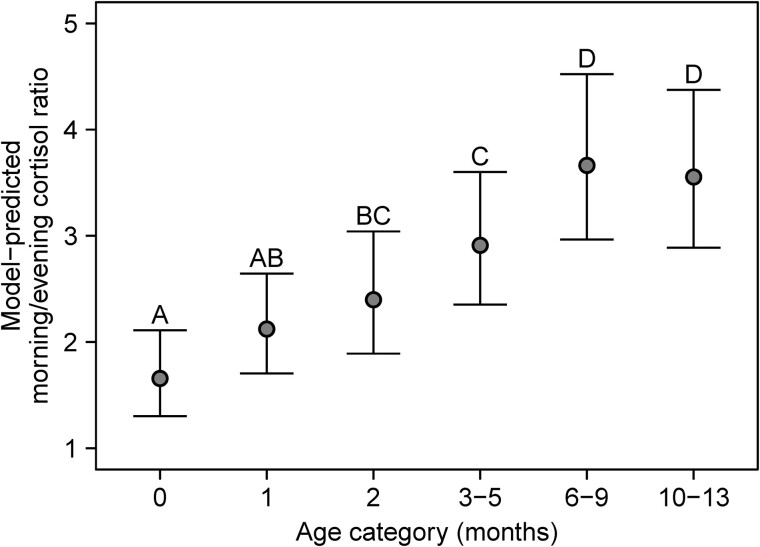
Ratio between morning and evening cortisol concentrations across different age categories in early infancy. Data points are shown as estimated marginal means and 95% CI. For all age categories with the same letter, the difference in the morning/evening cortisol ratio is not statistically significant (derived from interaction contrasts; *P* ≥ .05; adjusted by Sidak's method). If 2 age categories have different letters, the difference between the morning/evening cortisol ratio is statistically significant (*P* < .05; adjusted by Sidak's method).

In the main analysis, 14 985 observations were included (87.7% of all available observations) to assess the difference in cortisol concentrations between samples collected in the morning (between 06:00 and 12:00) and the evening (between 18:00 and 00:00). In the final model, the full random effect structure was retained as simplification significantly worsened the fit of the model (all *P* < .0001, likelihood ratio tests), but sex was removed as a covariate as inclusion did not significantly improve the model fit (χ² (1) = .598, *P* = .439, likelihood ratio test). A significant interaction between age category and time of day (categorized as morning vs evening) was observed (F[5, 6630] = 34.2; *P* < .0001), indicating that the difference between morning and evening cortisol concentrations depends on age. Post hoc pairwise comparisons indicated that morning cortisol concentrations were significantly higher than evening cortisol concentrations at each age category (Fig. 1). However, a significant increase in morning/evening cortisol ratio was observed with increasing age, showing the development of the cortisol rhythm as infants get older (Fig. 2). While at birth (ie, month 0) the morning/evening ratio was 1.7 (95% CI: 1.3-2.1), this increased to 3.7 (95% CI: 3.0-4.5) by month 6-9 and was similarly high (3.6, 95% CI: 2.9-4.4) at month 10-13.

Sensitivity analysis revealed that the between-study heterogeneity was limited (Supplemental Figure S3 (10)). For each hold-out cohort, the interaction between age category and time of day (morning vs evening) remained statistically significant (all *P* < .0001). In addition, the increase in the ratio between morning and evening cortisol concentrations across age was similar regardless of which study was omitted (Supplemental Figure S3 (10)).

**Figure 3.**
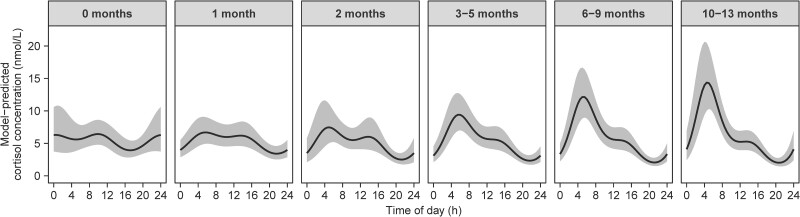
Model-predicted 24-hour rhythms in salivary cortisol concentrations across different age categories in early infancy. Data points are derived from the two-harmonic cosinor model (back-transformed from the log scale) and shown as estimated marginal means and 95% CI.

To further explore the development of 24-hour rhythmicity in salivary cortisol concentration, a cosinor model was fit to all available observations (n = 17 079). The full model containing 2 harmonic cosinor components with periodicities of 24 and 12 hours, age category as an interaction term, and participant and study included as random effects, provided a significantly better fit than simplified models with only a 24-hour component, no cosinor components, or no interaction with age (all *P* < .0001). This indicates that the 24-hour rhythm in cortisol concentrations changes with age during early infancy. Model-predicted cosinor curves show that the morning cortisol peak becomes more pronounced with increasing age (Fig. 3), corroborating the findings of the main analysis.

## Discussion

In our IPD meta-analysis, we found that the early-morning peak in cortisol secretion gradually emerges from birth onwards to form a stable morning/evening ratio from age 6-9 months. Robustness of our findings was demonstrated by sensitivity analysis. Exploratory analysis using cosinor modeling showed the emergence of a clear 24-hour rhythm with a single early morning peak during the second half of the first year of life.

For the first time, by using a meta-analytic approach the timing of the appearance of the early-morning peak of cortisol secretion was clearly pinpointed. Typical approaches of collecting this evidence from single studies have resulted in limitations in sample size, the number of age categories studied and/or the number of observations per 24-hour cycle. By combining individual participant data from 15 cohorts, we were able to produce robust findings.

Previous evidence has clearly shown that in mammals, the circadian timing system already develops prenatally (68). In primates, the suprachiasmatic nucleus (SCN) may already be present at mid-gestation, as ascertained by melatonin and dopamine D1 receptor labeling (69, 70). On the other hand, the vasopressin and vasoactive intestinal polypeptide cell population of the SCN for the major part only develops postnatally. Indeed, progressive maturation of circadian outputs is only observed after birth, with a pronounced rhythm in sleep/wake activity generally developing after age 2 months (71). A daily melatonin rhythm has been reported at age 12 weeks (72), and daily rhythms in cortisol generally between ages 3 and 6 months (4). Circadian rhythms for a variety of other hormones have been described with advancing age (73). The current data show that a significant morning/evening difference is already present in the first month of age. Although day/night differences in activity have already been reported in the first week after birth, consolidated periods of activity and rest only become apparent after 1 to 2 months of age (71). The development of the cortisol rhythm as presented here very much resembles that of the circadian rhythm of body temperature, with a significant rhythm being present at age 1 month, but with an increased amplitude at 3 months and an adult-like amplitude from 6 months onward (74).

A strong understanding of normative developmental changes in cortisol rhythmicity during infancy provides greater context for understanding deviations from the norm. Our findings, demonstrating that morning salivary cortisol is generally lower than evening salivary cortisol in young infants, emphasize the importance of developing age-specific reference values. As such, our results strongly suggest that previously published cutoffs for the diagnosis of adrenal insufficiency in adults (75) cannot be automatically extrapolated to infants.

In addition, our findings might have repercussions for the management of adrenal insufficiency in infancy. The cornerstone of hormonal replacement therapy is substitution of the missing hormone in a manner that is as physiological as possible. For this reason, older children with adrenal insufficiency, like adults with this condition, receive a morning hydrocortisone dose that is twice as high as the afternoon dose and the evening dose. However, such physiology-driven dosing is obviously more difficult for a hormonal axis that is highly dynamic and still developing, such as the infant HPA axis. Therefore, at present, there is no universally adopted recommendation for hydrocortisone replacement therapy across infancy. In practice, newborns receive 3 to 4 fixed doses of hydrocortisone per day, and, between ages 6 and 12 months, based on the discretion of the treating clinician, they switch to an adult-type scheme of hydrocortisone replacement therapy. However, although our findings may provide clues for hydrocortisone dosing in infants, several unresolved issues remain, such as a deeper understanding of infants' cortisol needs, hydrocortisone pharmacokinetics, availability of appropriate formulations, and, for congenital adrenal hyperplasia, strategies to decrease adrenal androgen production.

Data from an international registry of children with congenital adrenal hyperplasia showed that as early as after birth, body mass index SDS score started to increase, along with a high rate of blood pressure readings > 95th percentile (76), which may for an important part be explained by hydrocortisone overtreatment. It is likely that the knowledge gap in the normal development of HPA axis rhythmicity, in addition to other factors, such as fear for adrenal crises (77), has contributed to this overtreatment. Infants may be particularly vulnerable to hydrocortisone overtreatment, considering evidence suggesting that at an early stage of postnatal development metabolic set points are set for life (78). Indeed, there is overwhelming evidence from animal experiments demonstrating that glucocorticoid overexposure in early life leads to lifelong increases in fat mass, blood pressure, and glucose concentration (79).

Our data suggest that from early on, the morning dose should be gradually increased until it is twice as high as the afternoon and evening doses by 6 months of age to match the natural emergence of the 24-hour rhythm. However, we recommend that the efficacy of this strategy be tested experimentally prior to implementation, and that long-term outcomes be monitored.

The major strength of our study lies in the large number of observations for IPD meta-analysis (∼15 000 for all analyses). Furthermore, measurement of salivary cortisol, unlike cortisol in the bloodstream, carries the advantage that it is barely influenced by procedural stress. Limitations of our study include heterogeneity in the sampling and measurement of the data. Included studies differed considerably in the number of observations available for IPD meta-analysis (with one study (22) being responsible for half of all observations), in addition to differences in latitude, sampling protocols, collection methods, and assays. Moreover, differences in assay sensitivity, precision, and accuracy likely introduced noise in our analyses. However, findings remained virtually unchanged in leave-one-study-out sensitivity analysis, highlighting the robustness of our findings. Another limitation is that not all eligible studies were included, due to nonresponse or no access to the raw data. However, we were able to include the majority of available observations worldwide, and therefore it is unlikely that this limitation has materially influenced our results. Finally, all analyses in the current study were performed on a group level. The extent to which cortisol levels show a 24-hour rhythm in individual infants across different age ranges, as well as the degree of interindividual variability in the emergence of this rhythm, remain to be determined in carefully designed prospective studies.

In conclusion, the early-morning peak in cortisol secretion gradually emerges from birth onwards to form a stable morning/evening ratio from age 6-9 months. These findings advance understanding of the development of a key biological system for human functioning and have potential repercussions for hydrocortisone replacement therapy in infants with an inborn form of adrenal insufficiency. Nonetheless, a clinical trial addressing the long-term efficacy and safety of development-based hydrocortisone replacement therapy seems warranted prior to extension to clinical practice.

## Data Availability

Individual participant data were provided by the contributing studies based on the understanding that the relevant principal investigators will be contacted for further use of their data. Therefore, researchers who are interested in using these data can contact the principal investigators of each study included in this meta-analysis to request their individual participant data.

## Abbreviations

HPA: hypothalamic-pituitary-adrenal
IPD: individual participant data
